# Semantic and episodic processes differently predict false memories in the DRM task

**DOI:** 10.1038/s41598-023-50687-z

**Published:** 2024-01-02

**Authors:** Daniele Gatti, Luca Rinaldi, Giuliana Mazzoni, Tomaso Vecchi

**Affiliations:** 1 Department of Brain and Behavioral Sciences, University of Pavia, Piazza Botta 6, 27100 Pavia, Italy; 2 Cognitive Psychology Unit, IRCCS Mondino Foundation, Pavia, Italy; 3 Department of Health, Dynamic and Clinical Psychology, University La Sapienza, Rome, Italy; 4 Department of Psychology, University of Hull, Hull, UK

**Keywords:** Psychology, Human behaviour

## Abstract

There is a fervent debate about the processes underpinning false memories formation. Seminal theories have suggested that semantic memory would be involved in false memories production, while episodic memory would counter their formation. Yet, direct evidence corroborating such view is still lacking. Here, we tested this possibility by asking participants to perform the Deese–Roediger–McDermott (DRM) task, a typical false memory paradigm, in which they had to study lists of words and subsequently to recognize and distinguish them from new words (i.e., the false memory items). The same participants were also required to perform a semantic task and an episodic-source memory task. Our results showed that a higher number of false memories in the DRM task occurred for those participants with better semantic memory abilities, while a lower number of false memories occurred for participants with better episodic abilities. These findings support a key role of semantic processes in false memory formation and, more generally, help clarify the specific contribution of different memory systems to false recognitions.

## Introduction

Human memory cannot be simply conceived as a recorder that passively encodes, stores and faithfully retrieves information, but rather as a system that actively reconstructs information from memory traces^1,2^. Indeed, beyond accurate remembering, it has been shown that humans use their semantic memory to encode, store and remember information adapting it to their own previous knowledge, favoring consequently a gist extraction: in such a process, memory distortions and false memories may thus occur^3,4^.

One of the most widely used task to create false memories is the Deese–Roediger–McDermott task (DRM;^5,6^). During the DRM task, participants are first asked to encode several words divided in lists (i.e., within each list, the words are semantically/associatively related to a non-shown target word, named critical lure; e.g., *table*, *sit*, *legs*, *seat*, *couch*, *desk*, etc.—critical lure: *chair*) and then, after a brief distracting task, participants are asked to perform a recognition task (i.e., they have to indicate whether a given word was part of the memorized lists or not). Typically, during the recognition task, participants report as “old” (i.e., as previously memorized) a fairly large number of critical lures, although these words have not been presented before (for a review see,^7^).

The two main theories proposed to explain participants’ performance in the DRM task have traced back false memories occurrence to semantic processing. In particular, according to the activation-monitoring framework (AMF—^8,9^), the critical lure would be associatively hyperactivated by the presentation of the studied words (i.e., spreading activation), leading to high levels of false recognitions. Alternatively, according to the fuzzy-trace theory (FTT—^10,11^), participants would encode two different memory traces: a trace linked to episodic and perceptive features of the studied items, called verbatim trace, and a trace linked to the semantic content of each list, called gist trace, which would be responsible for the production of the false memories. Besides predicting a semantic basis for false memory production, both theories also predict that adequate source or episodic memory processes can reduce the occurrence false recognitions. That is, according to the AMF, if participants can successfully distinguish between words presented and words hyperactivated but not presented, the production of false memories would decrease^7^. Alternatively, according to the FTT, the verbatim trace would be involved in the correct rejection of non-presented words, since no episodic memory trace is available for those stimuli^10,11^.

In line with these theoretical perspectives, it has been shown that false memory occurrence can be predicted on a semantic and associative basis^9,12^. In particular, the backward associative strength (BAS, i.e., the association strength from the words that compose each list to the critical lure) is thought to be the central factor in determining false recall and false recognition^9^. Furthermore, recent studies also showed that the semantic component that underlies false memories can be decomposed into various sub-components^13^ and that higher false recognition rates occur for new words with higher semantic similarity with the ones studied^14–16^. At the same time, it has been shown that inducing a higher monitoring process warning participant about the false memory effect in the DRM^17^ or instructing them to focus on the distinctive aspects of each presented word^18^ would reduce false recognitions, thus pointing to the possible opponent role of episodic memory processes in countering memory distortions. Yet, the experimental manipulations introduced by prior research do not fully clarify the extent to which semantic and episodic memory processes differently contribute to false memories.

Previous studies have been indeed able to predict participants’ memory performance in the DRM task (e.g.,^9^, but see also:^19–21^), but this evidence is mainly limited to manipulations at the item level (e.g., the type of stimuli used) and to one component (i.e., episodic or semantic) tested at a time. Here, to directly probe the role of semantic and episodic processes in memory distortions and to include both components in the same model, we adopted an individual differences approach. An advantage of an individual differences framework is that it allows us to conceive individuals along continuous dimensions reflecting their semantic and episodic memory abilities, and to relate such natural variabilities with false memories production. Additionally, it allows to test episodic and semantic components together as they are related to participants, rather than items characteristics.

To give a broad overview, such an approach has proven to be successful in demonstrating that that higher working memory abilities^22–24^ or high memory self-efficacy^25^ are associated with a lower occurrence of false memories. Similarly, age differences have been linked to false memories, with false memories increasing in older individuals (e.g.,^26–29^; but cfr. also:^30^). Moreover, other studies investigating the link between creativity abilities such as convergent and divergent thinking have shown that the former, but not the latter, is associated with increased false memories^31^. Additionally, individual differences in need for cognition, an index that characterizes individuals’ preferences for engaging in effortful information processing^32^, can predict memory performance in the DRM task: in particular, participants with a high need for cognition, thus more likely to engage in effortful information processing, typically show a greater occurrence of false recalls^33^ and false recognitions^34^. Finally, other studies highlighted that false memory increases with age^35^, that DRM effects that are observed in adults are typically absent in young children and in learning-disabled children^36^ and that fewer false memories are made in between-languages conditions than within-language conditions at early age in bilingual children^37,38^.

Here, following the main theoretical accounts linking false memories with semantic and episodic processes, we aim to systematically dissociate the possible contribution of each memory system to memory distortions. We thus expect participants’ false recognitions of the critical lures to be positively associated with their semantic abilities (i.e., higher number of false memories for individuals with better semantic memory abilities) and negatively related with their episodic abilities (i.e., lower number of false memories for individuals with better episodic memory abilities). In addition to this, since the FTT posits that the verbatim trace is linked to both episodic and perceptive features of the studied items^10,11^, we also investigated participants’ source memory abilities. Participants were asked to perform a semantic task and an episodic-source memory task, from which we extracted the relative memory scores. We then predicted false and veridical memories occurrence in the DRM task using the scores extracted.

## Methods

### Participants

Sixty-four Italian right-handed students (23 males, *M* age = 23.89 years, *SD* = 3.18) participated in the study. All participants were native Italian speakers, had normal or corrected to normal vision and were naïve to the purpose of the study. Informed consent was obtained from all participants before the experiment. The protocol was approved by the psychological ethical committee of the University of Pavia and participants were treated in accordance with the Declaration of Helsinki.

### Stimuli and procedure

Participants were tested online using Psychopy^39–43^ through the online platform Pavlovia (https://pavlovia.org/). Participants performed the three tasks in three different days (i.e., the three sessions had to be completed within 1 week) at the same time of the day. The order of the tasks was counterbalanced across participants.

#### Semantic memory task

The task used was an associative/semantic priming task, in which participants were shown two stimuli (i.e., a prime stimulus and a target one, sequentially presented on the screen) and then were asked to judge if the second one (i.e., the target) was a word or a pseudoword. Generally, participants reaction times tend to be predicted by the degree of semantic relationship between prime and target words, with faster reaction times occurring for more related pairs. This speeding up is thought to reflect the enhanced involvement of semantic processing^44^.

Primes were 120 words selected from the Italian database provided by Montefinese et al.^45^. For each prime, a target word was chosen using the distributional semantic model SNAUT (^46,47^; http://meshugga.ugent.be/snaut-italian/), by selecting among the most semantically similar words and avoiding repetitions of the same target word across the task (for a review on distributional semantic models, see:^48^). Distributional semantic models have been shown to be high-performing across a wide range of semantic tasks (e.g.,^49^; and see also^50^), and they are equivalent to psychologically grounded associative learning models^46,48^. Critically, the semantic similarity index extracted from these databases is thought to involve both associative and semantic processes (for a similar approach with the DRM task, see^14–16^). Indeed, while some studies only found limited evidence relating DSMs indexes and associative processing^51^, others did report positive relationships using asymmetrical similarity measures as extracted from DSMs, which are thought to be more adequate for free associations tasks^52,53^. Then, the 120 word-pairs obtained were divided into two sets of 60 word-pairs (i.e., related and unrelated). In the unrelated set, primes and targets were pseudo-randomly mixed in order to remove the semantic link between the words (i.e., characterizing in turn the related set). From the same Italian database 120 additional words were extracted and transformed into pseudowords (i.e., reversing two letters: *paper*–*pepar*). All the pseudowords were readable.

Each prime appeared twice, one time followed by a word, and another time by a pseudoword. Related and unrelated pairs were comparable in terms of length and logarithm of the frequency of the first and second word, as well as in total length of the two words paired together and their paired logarithm of the frequency (all *p*s > 0.44, all BFs < 0.24).

Participants were shown two letter strings stimuli presented sequentially one after the other, and were required to judge if the second stimulus was a word or not. Participants were instructed to silently read the first letter string and to respond only to the second one as fast and as accurately as possible by pressing the left/right key (A and L) using the left and right index fingers, respectively; the response keys were counterbalanced across participants. The trials were shown in random order.

**Figure 1:**
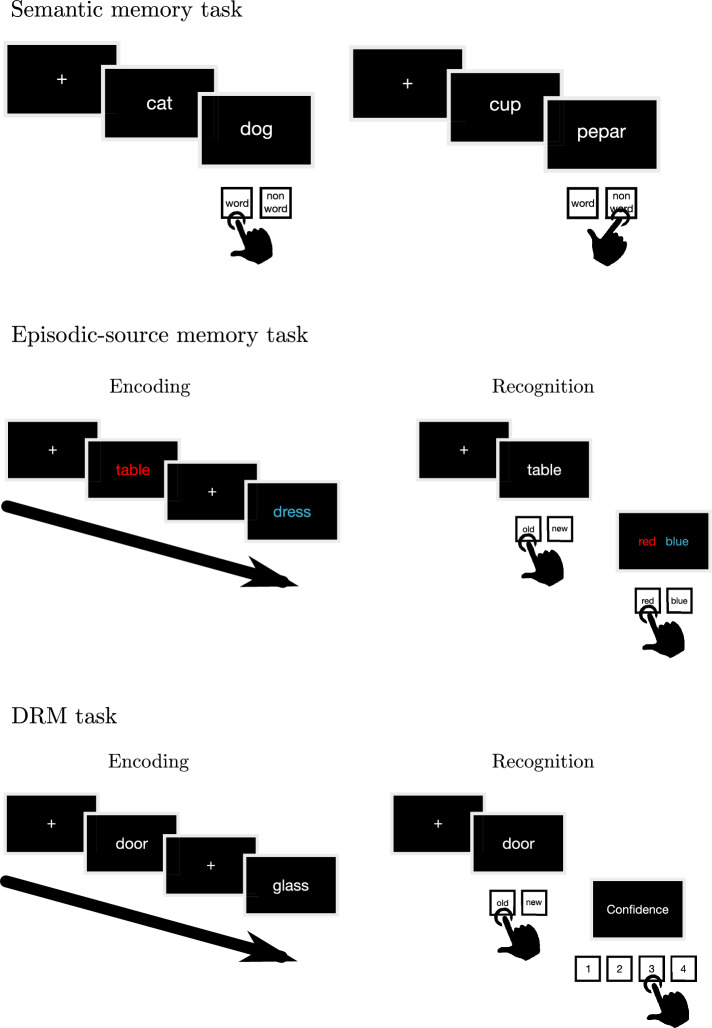
Schematic representation of the semantic priming task used to measure participants’ semantic abilities (top), of the task used to measure participants’ episodic and source memory abilities (middle), and of the two phases of the DRM task (bottom).

Each trial started with a central fixation cross (presented for 500 ms) and was followed by a first word (presented for 200 ms) and then by a second word (presented for 500 ms). Participants’ response ended the trial and moved to the fixation cross of the next trial. See Fig. 1 for a schematic representation of the semantic memory task.

#### Episodic-source memory task

We used an episodic-source memory task similar to the one recently employed by^54^. In a first phase, participants were asked to study several words printed in different colors and then, after a distracting task, to discriminate old/new words and to retrieve the color in which they have been originally shown. This task is thought to reflect general episodic abilities (i.e., the old/new judgements) and deeper source-memory processing (i.e., the color recognition).

We selected 100 concrete words from the Italian database provided by^55^. The 100 words were divided into two sets of 50 words. The first 50 were divided into two subsets, presented respectively in red and blue fonts. These words were presented during the memorization phase and were therefore “old” items in the subsequent recognition task. The remaining 50 words were not shown in the study phase and were used as “new” items in the subsequent recognition phase.

The two sets (i.e., as well as the two subsets) were comparable in terms of concreteness, imageability, familiarity, age of acquisition, context availability, abstractness, mode of acquisition, number of letters and logarithm of the frequency (all *p*s > 0.32, all BFs < 0.33).

During the first part of the task (i.e., encoding phase), participants were instructed to memorize 50 words: 25 words were colored in red and 25 words were colored in blue. Each trial started with a central fixation cross (presented for 500 ms) followed by a word (presented for 1500 ms) presented in either red or blue, followed by a blank screen (presented for 300 ms), then the script moved automatically to the next fixation cross. The words were shown in random order. At the end of the study phase, participants were required to perform an attention task (i.e., a modified version of the go-no go task) as a brief distracting task for 2 min.

Then participants had to complete the recognition phase. Participants were instructed to make old/new judgments and to respond as fast and as accurately as possible by pressing the left/right keys (A and L); the response keys were counterbalanced across participants. In case of “old” judgements (i.e., when participants judged the word as already presented in the memorization phase), the script moved to the next screen showing two question marks (one in the left side and one in the right side, one in red and the other in blue, with the position counterbalanced across participants); the participants were asked to make a source judgment by identifying the correct color in which the word was presented in the memorization phase. On the contrary, in case of a “new” judgement, participants were instructed to press the space bar, then the script moved automatically to the next trial. The trials were shown in random order. In this phase, each trial started with a central fixation cross (presented for 1000 ms) followed by a word (presented for 2500 ms) and, after participants’ response, by the source judgement. See Fig. 1 for a schematic representation of the episodic-source memory task.

#### DRM task

We used a typical version of the DRM task^6^. In a first phase, participants were asked to memorize several words grouped by lists (within each list, the words were semantically/associatively related to a non-shown target word, named critical lure; see below) and then, after a distracting task, to discriminate old/new words. Typically, participants report as “old” (i.e., as previously memorized) a large number of critical lures, although they were not in the studied items.

We selected 10 lists of words from the normative data for Italian DRM reported by^56^. Each list was composed by 15 words associatively related to a non-shown word (called *critical lure*). In this study, the words in position 13 and 14 were excluded and used as weakly related lures during the recognition phase, while the 15th word was not used; thus, participants studied 12 words lists. From the remaining lists reported by^56^—the ones not used in the present study—we extracted the words used as control stimuli.

The recognition phase was composed of 80 words, half of which had been studied and half of which had not. The 40 studied words presented in the recognition phase were those in positions 1, 4, 7 and 10 in the studied lists. Of the 40 non-studied words, 10 were the critical lures of the studied lists, 20 were weakly related lures, and 10 were unrelated words. The unrelated words were chosen randomly from the above-mentioned excluded lists. For example, for the list with *slow* as critical lure, the words included in the list were: *fast, snail, trend, dance, train, adagio, elderly, calm, delay, waltz, clear and tortoise*; the words used in the recognition phase as studied words were: *fast, dance, elderly and waltz*; the words used as weakly related lures were *peaceful* and *ant*; the unrelated word was *lemon*.

During the first part of the task, participants had to memorize a series of words (i.e., they were required to study 10 lists of words without interruptions). Participants were shown the 12 words that composed each of the 10 lists in descending forward associative strength (FAS; i.e., the association strength from the critical lure to the word that compose the list). The order by which the lists were presented was random, while the order of the words within each list was fixed (see Roediger and McDermott^6^). Each trial started with a central fixation cross (presented for 500 ms) followed by a word (presented for 1500 ms) and a blank screen (presented for 300 ms), then the script moved automatically to the next fixation cross.

At the end of the encoding phase, participants were required to perform an attention task (i.e., a modified version of the go-no go, different from the one employed during the episodic task) as a distracting task for 2 min.

Then participants were asked to perform the recognition phase. Participants were instructed to make old/new judgments and to respond as fast and accurately as possible by pressing the left/right key (A and L) using both hands; the response keys were counterbalanced across participants. After the old/new judgment, the script moved to the next screen and participants were asked to make a confidence judgement about their response using the keys 1, 2, 3, and 4 (i.e., with the key 1 representing the lowest levels of confidence and the key 4 representing the highest levels of confidence). The trials were shown in random order.

Each trial started with a central fixation cross (presented for 1000 ms) followed by a word (presented for 2500 ms) and then by the confidence judgement. The confidence judgement ended the trial and the fixation cross of the next trial was presented. See Fig. 1 for a schematic representation of the DRM task.

### Computation of the memory components scores

Signal detection theory measures were calculated using R-Studio^57^, by means of the *psycho* package^58^. (Note that in the *psycho R* package, adjustments for extreme values are made following the recommendations of^86,87^.)

The semantic memory score was computed as the priming effect z-transformed score (i.e., in order to get homogeneous values from different memory scores used as predictors in the final analyses) induced by the prime on the processing of the target word (i.e., the speeding up in participants’ correct reaction times; RTs). That is, for each participant, the semantic score was computed by subtracting the mean RTs for related prime-target words pairs to the mean RTs for unrelated prime-target words pairs (i.e., positive values indicate a facilitation in the processing of the target word as induced by the related prime as compared to the unrelated prime and, consequently, a stronger activation of the semantic memory network). In analogy with previous studies using associative and semantic priming tasks^58^, we analyzed responses with RTs < 1200 ms (5% of the trials excluded), since slower responses are thought to reflect distraction or low familiarity with the words showed, rather than lexical access^59^. Similarly, responses judged too fast (RTs < 300 ms) were excluded (2% of the trials excluded). Critically, and as expected, participants’ RTs for related words were overall significantly faster compared to RTs for unrelated words, *t*(63) = 2.36, *p* = 0.02, thus reflecting a facilitation due to semantic relatedness.

The episodic memory score was computed as participants’ *d’*^60^, that is, the z-value of the hit-rate (“yes” response when the correct response is “yes”) minus the z-value of the false-alarm rate (“yes” response when the correct response is “no”) in old/new judgements of the episodic task. Participants’ *d’* was computed only on responses with RTs > 300 ms and RTs < 3000 ms (6% of the trials excluded).

The source memory score was computed as participants’ *d’* in the source judgment of the episodic task. In this case, we considered only trials in which participants responded “old” to old words (i.e., only trials in which there was actually a color source to be accessed to), since including also the other items would have caused an overestimation in participants’ error rate. To compute the source memory score, we considered as hits those trials in which participants correctly identified the correct color, while we considered as false alarms those in which participants indicated the wrong color. We included only trials with RTs > 150 ms and RTs < 3000 ms (2% of the trials excluded).

The semantic memory score was not correlated with the episodic memory score, *r* = − 0.09, *p* = 0.46, nor with the source memory score, *r* = − 0.11, *p* = 0.39. The correlation between episodic memory score and the source memory score was higher but not significant, *r* = 0.23, *p* = 0.07. The correlation between episodic and source memory scores is consistent with the theoretical view according to which they would represent two sub-components of the same memory system^61^.

Finally, in the DRM task, trials with RTs < 300 ms and RTs > 3000 ms were excluded from the analyses (4% of the trials excluded for old/new judgements; 11% of the trials excluded for confidence judgements). The analyses on confidence judgements were performed including only the trials in which participants responded “old” (i.e., hits for studied words and false alarms for critical lures, weakly related lures and unrelated words).

### Data analysis

All the analyses were performed using R-Studio^57^. Data were analyzed through a mixed-effects approach, which incorporate both fixed-effects and random-effects (i.e., associated to statistical units as participants and task stimuli) and provide more detailed information about relationships among predictors and outcome variables compared with Pearson correlation (which simply measures the strength of the linear relationship between each selected pair of variables independent of the others;^62^). Generalized linear mixed models (GLMMs) were run using the *lme4* package^63^, while cumulative link mixed models (CLMMs) were run using the *ordinal* package^64^. The graphs reported were obtained using the *effects* package^65,66^.

First, we explored the memory processes subserving false and veridical memory. We ran a GLMM having old/new judgements in the DRM task (i.e., “new” responses were scored as 0, whereas “old” responses as 1) as the dependent variable and subjects and items as random intercepts. In particular, in our statistical model, we additively included as predictors the z-transformed semantic, episodic and source memory scores along with their interaction with the Type of stimuli (critical lures vs. weakly related lures vs. unrelated words vs. studied words). That is, in the *lme4* syntax, we tested the following model:

$$Response \sim \left( {Semantic + Episodic + Source} \right)*Type + \left( {1{|}Participant} \right) + (1|Item)$$

Second, we explored the memory processes subserving confidence judgements when making veridical and false memories. We ran a CLMM with confidence judgements (i.e., from low to high confidence, 1 vs. 2 vs. 3 vs. 4, as a factor) as the dependent variable and subjects and items as random intercepts. The semantic, episodic and source memory scores were additively included along with their interaction with the type of stimuli (critical lures vs. weakly related lures vs. unrelated words vs. studied words; i.e., the model is analogous to the one reported above for old/new judgements).

In the “Results” section we report only the analyses on old/new judgements, since no significant effect was found for confidence judgements (i.e., the results on confidence judgements are reported as Supplementary Materials). To further check for multicollinearity, we inspected the variance inflation factor (VIF) of each predictor. VIF indexes were estimated using the check_collinearity command from the R package *performance* (Lüdecke et al.^68^). VIF has 1 as lower boundary indicating no collinearity and has no upper boundary, with its interpretation being: the higher the value, the higher the collinearity. Here we adopted as threshold the one suggested by^67^: a VIF < 5 indicates a low correlation of that predictor with other predictors. While 5 < VIF < 10 indicates a moderate correlation, VIF > 10 indicates high, not tolerable correlation of model predictors.

## Results

The *Pseudo-R*^*2*^ (total) of the model estimated was = 0.46 and the *Pseudo-R*^*2*^ (marginal) was = 0.32; no multicollinearity issues were detected, all the VIFs < 3.2.

The effects of episodic memory score, *χ*^*2*^(1) = 5.41, *p* = 0.02, semantic memory score, *χ*^*2*^(1) = 4.79, *p* = 0.03 and type of stimuli, *χ*^*2*^(1) = 223.75, *p* < 0.001, were significant. Conversely, the effect of source memory score was not significant, *χ*^*2*^(1) = 1.49, *p* = 0.22. Critically, the interactions semantic memory score by type of stimuli and episodic memory score by type of stimuli were found to be significant, respectively, *χ*^*2*^(3) = 10.83, *p* = 0.01; *χ*^*2*^(3) = 24.22, *p* < 0.001.

**Figure 2:**
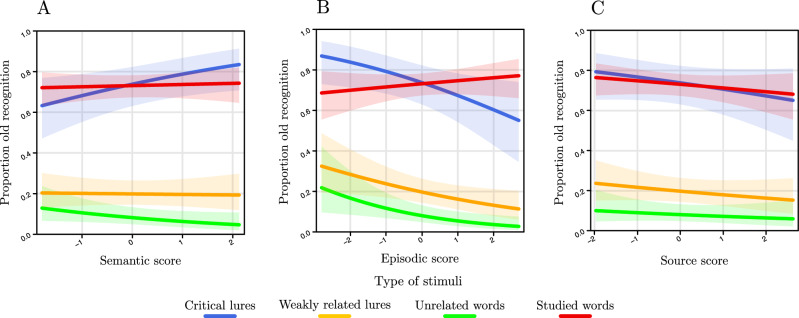
Results from the GLMM including the interaction type of stimuli by semantic memory score (**A**), episodic memory score (**B**) and memory source score (**C**) as predictors and the proportion of “old” responses (divided in critical lures, weakly related lures, unrelated words and studied words) as dependent variable. The results show that the occurrence of false recognitions of the critical lures is predicted by both semantic and episodic memory scores, albeit with different directions (i.e., more false memories for individuals with better semantic memory abilities; less false memories for individuals with better episodic abilities). On the contrary, the source memory score does not have any effect on false memories.

The significant interaction semantic memory score by type of stimuli (Fig. 2A) indicates that for critical lures the higher the semantic memory score, the higher the chance of making false memories, *z* = 2.19, *p* = 0.03. No effect was found for studied words, *z* = 0.33, *p* = 0.74, weakly related lures, *z* = − 0.16, *p* = 0.87, nor for unrelated words, *z* = − 1.77, *p* = 0.08.

The significant interaction episodic memory score by type of stimuli (Fig. 2B) indicates that for critical lures, *z* = − 2.33, *p* = 0.02, weakly related lures, *z* = − 2.25, *p* = 0.02, and unrelated words, *z* = − 2.62, *p* = 0.01, the higher the episodic memory score, the lower the chance of making false memories. No effect was found for studied words, *z* = 0.88, *p* = 0.38.

Conversely, the interaction source memory score by type of stimuli was not significant, *χ*^*2*^(3) = 0.36, *p* = 0.94 (Fig. 2C).

## Discussion

In the present study, we investigated individual differences in memory processes subserving false recognitions in a classic DRM task. In particular, we assessed participants’ semantic, episodic and source memory abilities and explored whether these three memory components differently predicted performance in the DRM task. Our findings indicate that the occurrence of false memories is predicted by both semantic and episodic memory, yet to a different extent. That is, higher semantic memory abilities predicted a *larger* number of false memories, while higher episodic abilities predicted a *smaller* number of false memories. Interestingly, the effect of semantic memory was specific for critical lures (i.e., better semantic memory abilities were associated with an increased likelihood of false recognitions), while the effect of episodic memory was found for all the three types of new stimuli, namely critical lures, weakly related lures and unrelated words (i.e., better episodic memory abilities were associated with a reduced likelihood of memory errors).

These findings have potential implications for our understanding of false memories formation. First, previous studies investigated semantic memory involvement in the DRM task only at the item level^9^. That is, these studies manipulated the level of semantic memory involvement during the DRM task, by including word lists that could be differently associated with a critical lure (i.e., hence having lists more semantically related with critical lures,^12^). Typically, the more the word lists are semantically related with the critical lure, the higher the occurrence of false memories^9,12–16^. Our findings, by showing that also differences in participants’ semantic abilities play a role in the formation of false memories, indicate that these two components (i.e., the semantic content of the words processed and the extent to which participants rely on semantic memory) are both active during the DRM task. Second, by showing that also participants’ episodic abilities contribute to false memories, our data suggest that episodic memory is actively involved while judging if a word was studied or not. Such an effect is likely reflecting the recall-to-reject process^68^ in its diagnostic component^69^. That is, in the recognition phase, participants would recall the words studied and compare them with the ones actually presented: if the episodic trace for the word actually presented is weaker compared to the ones studied, the participant is less likely to accept the word as “old”^69,70^. Notably, the contribution of episodic processes would be hard to be operationalized at the item level, again testifying the advantages of the methodological approach employed here.

Conversely, source memory—the participants’ ability to retrieve the exact perceptive features (i.e., the color of the studied words)—did not have any impact on the DRM task. These findings, indicate that source memory abilities (at least in the way we operationalized them, but for an alternative perspective on source memory see:^71^) might not play a major role in true and false memories formation. To account for this null finding, we note that in the DRM task the studied words are typically presented from the same source (e.g., in the same ink color or read aloud by the same experimenter). In the studies in which the source of the information was manipulated (e.g., which experimenter read the study list;^39^; but see also:^72^) participants certainly noticed the difference, but this did not substantially affect the rate of false memories reported. Our operationalization of source memory ability thus may tap on aspects that are not related to the specific cognitive requirements of the DRM task. Additionally, the lack of a source memory involvement in correctly rejecting new stimuli could also be specifically related to the fact that here both the encoding and recognition phases were presented in the visual modality (while a mixed auditory-visual modality has been employed in some previous research, e.g.,^6^). That is, the sensory facilitation occurring in the visual modality could have induced participants to rely on a lesser extent on source memory.

More generally, these data support the utility of individual differences for investigating the factors involved in false memory production and formation. Previous studies have indeed investigated participants’ performance in the DRM task using an individual differences approach showing that several variables underly some of the individual variation in susceptibility to memory illusions. For example, participants’ performance in the DRM task has been predicted from working memory abilities^22–24^, age^24,27^, memory self-efficacy^25^, creativity^31^, or need for cognition^33,34^ Here, for the first time, we employed individuals’ semantic and episodic memory to predict responses in the DRM task. Our results support the specific contribution of episodic and semantic processes in the production of false memories, thus corroborating previous theoretical evidence accounting for the DRM task.

In particular, these findings directly support the two mainstream theories accounting for false memory, the AMF and FTT theories, which predict the occurrence of false recognitions on an associative/semantic basis, while adequate episodic memory processes should counter them^8–11^. The main difference between AMF and FTT is that the former assumes that critical lures are falsely recognized due to the associative link between that and the list words, while according to the latter theory critical lures and list words would share semantic features that underlie false recognition. Yet, disentangling the associative from the semantic framework may be sometimes difficult, as often two words that are associatively related are also semantically related (^73,74^; but see^12^). This is also reflected in the task used here to assess semantic memory, which necessarily involves both semantic and associative processes and was selected on purpose to be representative for both AMF and FTT hypotheses.

On the one hand, according to the AMF, during the encoding phase, the presentation of the list words would associatively hyperactivate the critical lure^8,9^. Consistent with this possibility, Roediger et al.^9^ have shown that the more the list words are associatively related to the critical lure, the more the participants are prone to false memories and recognitions. Crucially, the AMF hypothesis also posits that, during the recognition phase, successful monitoring processes (i.e., the ability to distinguish whether retrieved information refers to past events or not;^75^) can counteract false recognitions and enhance veridical memories^8,9^. In line with this possibility, here we showed that the higher the participants’ episodic memory ability, the lower the chances of making false memories. Critically, this effect was not limited to critical lures, but spread as well to other types of new words (i.e., weakly related lures and unrelated words), indicating that participants could adopt the same strategies in rejecting the various types of new stimuli.

On the other hand, according to the FTT participants’ false memories would rely on a semantic trace, called gist trace and linked to the semantic content of each list^10,11^. Such a theoretical framework is as well in line with our findings: participants with higher semantic memory abilities are thought to have stronger abilities in forming the gist trace, resulting in turn in a higher false recognition occurrence. Furthermore, according to the FTT, correct rejections would depend on the verbatim trace, linked to both episodic and perceptive features of the studied words^10,11^. Here, however, we could only find evidence for a relationship between episodic memories and false recognitions, but not with source memory (i.e., defined in our study as the ability to correctly retrieve the perceptual features of studied words).

It should also be noted that the lack of source memory effects speaks against several major assumptions of these two theories. However, the operationalization of this memory component may have partially determined this pattern of results. That is, the yes/no judgement as measured in the episodic component of the task includes by itself certain source memory components (e.g., indicating the color in which the word was presented in the memorization phase), as indeed testified by the moderate correlation (*r* = 0.23) between the two indexes. Following this line of reasoning, one may argue that the episodic memory score, including both components, could be more comprehensive. Note also that the two memory processes—memory judgements in the DRM and episodic recognition—were similarly assessed here, with the request to judge whether an item was studied or not in both cases. Although contextual processing is intrinsically part of the episodic memory construct, the episodic task used here can be thought of as indexing both episodic and source memory components.

Beyond the two main theoretical hypotheses accounting for false memory, previous evidence suggests that false recognitions follow a continuous semantic gradient in terms of backward associative strength^9^ and semantic similarity^14–16^ between the studied words and the critical lures. Similarly, it has been shown that the semantic component that should elicit false recognitions can be decomposed into various sub-components (situation features, synonyms, antonyms, and taxonomic relations;^13^). The results of the present study extend these findings by showing that also individual differences in semantic and episodic memory play a crucial role in determining false memory production. These results also point out the need for investigating semantic and episodic processes while analyzing participants’ performance in the DRM task in order to link false recognition proportion to episodic vs. semantic modulation. For example, employing this approach in neuromodulation studies would allow for a better comprehension of the underlying memory processes also on a neural level (i.e., if enhanced false recognition depends on impaired episodic memory on enhanced semantic memory; for alternative approaches see also:^16,76^).

Finally, one possible point of concern could be related to the lack of effects of semantic and episodic memory on veridical recognition. To account for this pattern of results, we first note that, besides unrelated words, we also used as control stimuli weakly related words; these weakly related words could have induced a shift in participants’ response criterion favoring in certain cases a conservative response bias (i.e., recognizing that also weakly related words were shown in the recognition task could have induced some participants to respond “no” more often to less semantically related studied words). Another possible explanation, on the episodic/source memory distinction, is that the task used here can only index the component explaining correct rejections, while other sub-components could be observed more specifically using other types of tasks. Similarly, regarding the semantic task, one may argue that the task adopted indexes the speed of activation but not its magnitude. Thus, the observed lack of effects for veridical memory could depend on this, as it is known that multiple (semantic) sub-components could be differently involved in the DRM task (^13^, and that participants’ semantic memory could be decomposed into different sub-systems involved in veridical and false recognitions. Another relevant factor could be the operationalization of the semantic score as well. There are certainly commonalities between the priming effect and the hyper-activation of the lure in the DRM task during the encoding phase^77–79^, but semantic involvement in veridical recognition could be indexed with more reliability through other tests. Indeed, multiple models have been proposed to describe semantic memory, each one mainly related to a specific set of tasks and behaviors^80^. The decision to employ a semantic priming task was driven by experimental evidence showing abnormal semantic priming effects in population of patients characterized by impaired semantic processing^81,82^, hence validating this task as tapping on semantic memory. However, extracting the semantic scores from other tasks could better account for veridical recognition (or participate in explaining false recognition), with this approach being particularly promising for future research in order to distinguish between the AMF and FTT theories.

Other possible points of concern could be related to the precise memory processes indexed by the task employed in the present study. That is, while we assumed that the semantic task primarily indexes semantic memory components and that the episodic one primarily indexes episodic components, they could also (partially) account for specific sub-components of the other. Indeed, while for example in the case of the episodic task the instructions did not emphasize to process the meaning of the words shown, we cannot exclude that the participants took advantage of words semantics while solving the task (even though the content of words shown was very variable); as such, the episodic index includes also some semantic components. The same argument can be applied to the semantic priming task. This is in line with recent theoretical perspectives supporting semantic and episodic memories as highly interdependent^83^, with this possibly being at play in our paradigms too and thus making it complex to further disentangle the two. That said, it should be noted that the semantic and episodic/source memory predictors were not correlated and thus, even though we cannot exclude some (limited) crossed effects, we can argue in favor to the fact that each task mainly indexes a specific component. Parallelly, it should also be noted that, while participants’ behavior in the DRM task and in the semantic priming task are quite different in terms of task requirements, this is not true for the episodic memory task, that varies from the DRM only in terms of material and source memory judgement. That is, recognition behavior, also in the context of the DRM task, can be based on the combination or relative contributions of recollection and familiarity (e.g.,^84^). These commonalities between the two tasks (possibly in terms of underlying recognition memory processes) could (partially) drive the observed episodic effects (which however do only involve correct rejections).

In conclusion, by taking an individual differences approach, in this study we show that various memory systems are differently involved in veridical and false memories production. Our findings thus point to the importance of investigating individual differences linked to false memory production, possibly through more ecological paradigms, since this has ultimately links with the quality of justice trials and witness evaluation^85^.

### Supplementary Information

Supplementary Information 1.

Supplementary Information 2.

## Data Availability

The data analyzed in this study are reported as Supplementary Materials.
