# Supplementary material for: Semantic and episodic processes differently predict false memories in the DRM task
Source: Sci Rep. 2024 Jan 2;14:256. doi: 10.1038/s41598-023-50687-z (PMC10761856; doi:10.1038/s41598-023-50687-z)
Supplement: Supplementary file 2 — Supplementary Information 2. [file 41598_2023_50687_MOESM2_ESM.docx]

**Supplementary Materials**

**Semantic and episodic processes differently predict false memories in the DRM task**

Daniele Gatti ^1^, Luca Rinaldi ^1^, Giuliana Mazzoni ^2,3^, & Tomaso Vecchi ^1,4^

*^1^Department of Brain and Behavioral Sciences, University of Pavia, Pavia, Italy*

*^2^Faculty of Medicine and Psychology, University La Sapienza, Rome, Italy*

*^3^School of Life Sciences, University of Hull, Hull, United Kingdom*

*^4^Cognitive Psychology Unit, IRCCS Mondino Foundation, Pavia, Italy*

**Confidence judgements**

The results of the CLMM on confidence judgements are reported in Figure S1. Besides the type of stimuli, *χ*(3) = 64.7 *p* < .001, no other significant main effects nor interactions were found.

**
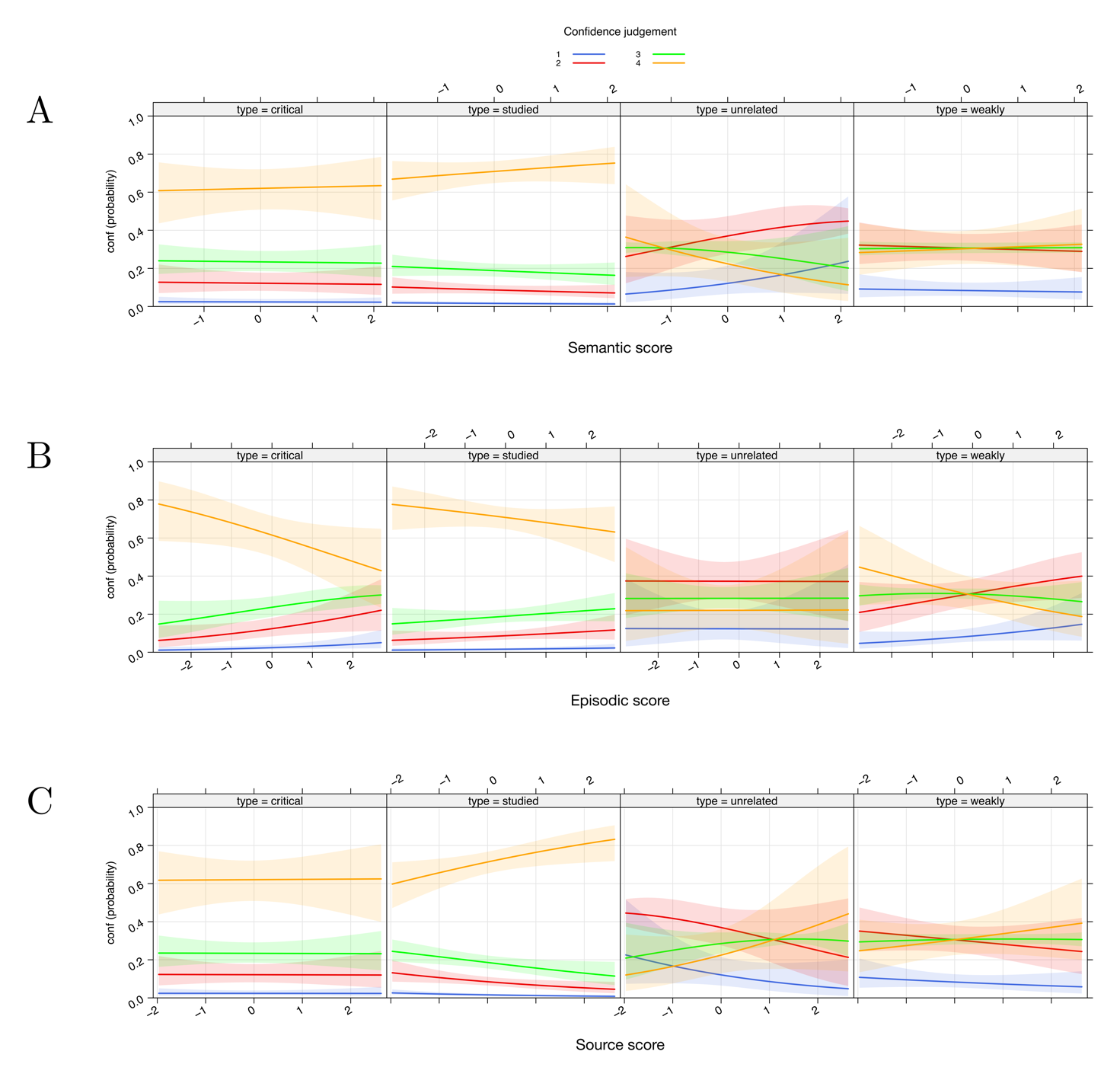
**

**Figure S1.** Results of the CLMM including the interaction type of stimuli by semantic memory score (A), episodic memory score (B) and memory source score (C). No significant interactions were found.
